# Rationalizing the Influence of Co‐Design on Distress, Clinical Decision‐Making and Disease Self‐Management of Cancer Patients‐as‐Partners: A Quasi‐Experimental Study

**DOI:** 10.1111/hex.14113

**Published:** 2024-06-14

**Authors:** Zahraa Alrayshouni, Alaa Dayekh, Ahmad El‐Tassi, Annamária Pakai

**Affiliations:** ^1^ Patients Affairs Department Saint George Hospital Beirut Lebanon; ^2^ Doctoral School of Health Sciences, Faculty of Health Sciences University of Pécs Pécs Hungary; ^3^ Nursing Department, Faculty of Health Sciences Beirut Arab University Beirut Lebanon

**Keywords:** cancer care, healthcare, hospital, patient engagement, patient participation, patient partnership, quality improvement

## Abstract

**Introduction:**

Cancer is regarded as a major worldwide burden. Patient distress has been linked to disease progression. Studies show that engagement strategies affect clinical decision‐making and patient outcomes. The optimal engagement method is a partnership that integrates the patient's expertise into the comprehensive co‐design of the healthcare system.

**Objectives:**

This is the first study to investigate cancer patient‐as‐partner experience and its impact on distress levels, decision‐making and self‐management.

**Methods:**

It is a quantitative and quasi‐experimental study that adopted a partnership committee at a Lebanese hospital. A stratified random sampling approach was used, and data were collected by self‐administered questionnaires. We utilized the standardized distress thermometer and PPEET.

**Results:**

We recruited 100 patient partners. Cancer patients‐as‐partners had optimal engagement experience in QI projects (mean = 4; SD = 0.4). The main partnership benefit was improved hospitalization experience (49%). Almost half of PP reported no challenges faced (49%). Recommendations for improvement were training (19%), team dynamics management (12%) and proper time allocation (7%). The distress level post‐partnership was significantly reduced (*t* = 12.57, *p* < 0.0001). This study highlights the importance of partnership and ‎its ability to influence shared decision‐making preference [*χ* ^2^(2) = 13.81, *p* = 0.025] and self‐management practices [*F*(3, 11.87) = 7.294, *p* = 0.005].

**Conclusion:**

Research findings suggest that partners from disadvantaged groups can have optimal partnership experience. A partnership model of care can shape the healthcare system into a people‐oriented culture. Further research is needed to explore diverse PP engagement methodologies and their effect on organizational development.

**Patient or Public Contribution:**

Patients and family members were engaged in the co‐design of the study methodology, especially the modification of a research instrument. Patient partners with lived experience were involved in the patient partnership committee as core members to improve healthcare system design and evaluation.

**Abbreviations**
HP: healthcare professionals
IRB: Institutional Review Board
PFPC: Patient and Family Partnership Committee
PP: patient partners
PPEET: Public and Patient Engagement Evaluation Tool
SGH: Saint George's Hospital

## 1 Introduction

Globally cancer is the second leading cause of death, after cardiovascular disease, and a significant barrier to raising life expectancy. It is regarded as a major worldwide burden. A 2020 report shows about 19.3 million additional incidences of cancer worldwide, along with approximately 10.0 million cancer‐related deaths [1]. Although cancer registries fluctuate based on the country's growth level, the incidence and mortality of cancer are declining in developed nations like the United States and Europe. On the other hand, the less developed and poorer nations are experiencing a rise in the prevalence of cancer [2]. In Lebanon, citizens face cancer as a challenge on the additional portion of the country's ongoing political and economic issues. Lebanon has the highest cancer incidence rate among Arab nations [3].

Cancer and its therapy are a significant source of stress, which makes it difficult for patients to adjust to the disease and causes emotions like worry or depression [4]. Available treatment approaches cause the body to change significantly and undergo severe changes, such as disfigurement, scarring, skin changes and loss of a bodily part, which can cause general body image problems [5]. Body image distress is a serious psychological condition that is caused by a noticeable deviation from one's ideal look and a functional impairment in it [6]. Studies have shown that self‐perception influences decision‐making. It provides a point of reference for values and motivations to guide important decisions and improves the evaluation of options by raising the decision‐maker's awareness of one's reactions [7]. Thus, oncology patient distress can directly affect the clinical outcomes and quality of care [1]. This has urged healthcare system designers and authorities to create means to maximize disease self‐management practices to achieve better clinical outcomes and fewer complications [8]. One way to mitigate distress complications and enhance the clinical self‐management of cancer patients is to reinforce proper patient education. It has been said that chemotherapy adherence is a multifaceted and ever‐changing process that necessitates constant observation, instruction and tailored follow‐up. Research indicates that providing personalized education and ongoing support to cancer patients improves their self‐efficacy [9]. Cumulative engagement policies will enhance shared decision‐making between the clinician and the patient. As a consequence, increased patient participation in decision‐making aligns with the optimization of the healthcare services value [10, 11].

The healthcare system design has witnessed multiphase evolutions over time aiming to enhance patient–physician communication [12]. Having multiple models of patient engagement methodologies in the clinical decision‐making process, as revealed by numerous studies [10, 11], the patient partnership model of care is by far considered the most innovative approach [13, 14, 15]. Its evidence‐based protocols have proven to enhance the quality of care, hospitalization experience, distress level and clinical management [13, 14]. However, the available scientific research has limited the focus on cancer management and cancer patients as partners within the partnership framework [14, 16, 17, 18, 19, 20, 21, 22, 23, 24, 25, 26, 27, 28, 29, 30, 31]. This study will demonstrate the experience of cancer patients as partners in quality improvement. We aim to prove the positive influence of the partnership model of care on therapeutic clinical decision‐making preferences, efficient self‐management practices and reduced cancer‐related distress.

## 2 Materials and Methods

### 2.1 Research Design and Setting

This quantitative, prospective, cross‐sectional mixed‐method study included 100 participants from Saint George's Hospital (SGH) between 1 July 2023 and 29 January 2024. It is categorized as an interventional study without a concurrent control approach since it includes partnership meetings to engage the patient partners (PP) recruited in the design, implementation and evaluation of the healthcare system. We conducted descriptive and analytical analyses on data collected from 100 PP.

### 2.2 Ethical Consideration

The IRBs of the corresponding institutions approved this study. The IRB ID number was 2023‐H‐168‐HS‐M‐0573. Written consent was obtained from the PFPC members who attended the meetings. Data were kept confidential and used for research purposes only. ‎

### 2.3 Participants

A stratified random sampling technique was used to select the sample that is most representative of the population [32]. The sampling frame was developed based on the large admission system database. The frame included patient status as inpatient hospitalization or outpatient ambulatory care, and type of care provided during hospital stay. We first specified the study setting and targeted patients admitted to this specific hospital. Second, we used the electronic database to divide the population into subpopulations based on the type of care, such as cancer patients, surgical patients and so on. Then, after dividing the cancer patients into strata based on well‐defined inclusion and exclusion characteristics, the research team randomly drew samples from each cancer patient group. Candidates from the lists were organized in alphabetical order and given a number by the admission system. The research team selected the participants to be contacted based on odd and even number choices mitigating the occurrence of sampling bias.

The sample number target was set to recruit a minimum of 120 PP computed from Slovin's formula *n* = *N*/(1 + *Ne* ^2^), where ‘*n*’ is the sample size, ‘*N*’ is the population size and ‘*e*’ is the margin of error set at 5%. However, we reviewed the existing literature to resemble the research plan [14, 16, 17, 18, 19, 20, 21, 22, 23, 24, 25, 26, 27, 28, 29, 30, 31] and to allow a significant comparison of research findings and found that our sample target is the highest. Out of 811 hospitalized patients, the admission system generated 362 patients that fit the sampling frame, and we contacted 260 candidates. We ended up recruiting only 103 patients and family members, who were eligible and motivated to participate. However, three patients were lost to follow‐up, where one patient died, another patient was transferred to ICU due to clinical deterioration and the third dropped out. PP, who lost to follow‐up, were excluded from data analysis. This type of interventional study justified the sample number selected as it included the research team's effort to select, screen, recruit, provide training and implement the complete phases of the partnership model of care. Thus, the sample size adapted to the nature of this study and the resources available, but most importantly it abided by the quantitative research guidelines, to drive conclusions that are validated, reliable and representative of the population [33].

The inclusion criteria for the PP were as follows: Patients diagnosed with cancer disease; Family members and representatives taking care of a cancer patient; Parents and legal representatives of a paediatric cancer patient; Inpatients and/or ambulatory hospitalization care patients; Being available and willing to allocate time and effort; and being treated at the same hospital. Participants were excluded if they were less than 18 years old; Critically ill with unstable health cancer patients; and lacked the time for involvement in this study. These assumptions used for successful implementation were carefully studied and compared with similar methodologies from the literature [14, 16, 17, 18, 19, 20, 21, 22, 23, 24, 25, 26, 27, 28, 29, 30, 31].

### 2.4 Process

#### 2.4.1 Pre‐Partnership Preparedness

PP were asked to sign a confidentiality agreement statement after they were recruited. The research team performed a preliminary assessment of the distress level through the standardized distress thermometer and problem list tool, which was created by the National Comprehensive Cancer Network, for all recruited partners [34]. These data acted as the baseline for the comparative analysis. Partners were engaged in a brief terminology training to create a knowledge base for the patients who participated, who are of diverse backgrounds and fluctuating educational levels.

#### 2.4.2 Partnership Intervention

The partnership committee in this hospital, which was launched by SGH in 2020 as the first official partnership model of care, included healthcare professionals of different specialties and years of experience along with patients recruited as partners as fixed members in this committee. The PFPC goal is to diagnose system failures, assess areas for improvement and co‐design, co‐implement and co‐evaluate quality initiatives.

Our research team invited partners to the meeting, after organizing the logistics required to facilitate the attendance of all parties. We have preserved an equal ratio of attendance among partners and healthcare. Overall we conducted 34 meetings, each meeting included three PPs and three HPs. Only one PP requested to participate in the partnership committee meeting alone based on her preference. The quality improvement topics were selected based on the members' preferences and by voting. Participants chose diverse QIP topics. Meetings took on average 40–60 min of discussion. The investigators documented meeting minutes and kept them in records. The hospital officials implemented the suggested quality improvement plan by the Patient and Family Partnership Committee. Finally, we provided feedback to the participants on the project implementation, facilitators, barriers and results. This favours the credibility of such a model of care and builds mutual trust among partners and providers.

#### 2.4.3 Post‐Partnership Evaluation

We evaluated partners' experience directly after the implementation of the partnership model of care through a standardized Public and Patient Engagement Evaluation Tool (PPEET) self‐administered survey, which was developed in 2018 by Julia Abelson and the PPEET Research‐Practice Collaborative at McMaster University [35]. Also, after three business days of the project implementation, the investigators re‐assessed distress levels and the impact of partnership initiatives on the patient's outcomes, clinical decision preferences and self‐management initiatives using a well‐structured piloted and statistically tested self‐administered questionnaire.

### 2.5 Data Collection

We initially gathered data through the administration of the Patient Partner Characteristics Survey, which encompassed patient demographics, personal characteristics, disease characteristics and therapeutic plans. Subsequently, we assessed distress levels by means of direct surveys utilizing the National Comprehensive Cancer Network (NCCN) instrument, a self‐reported self‐assessment tool that employs a 0‐to‐10 rating scale [34]. Following the completion of PFPC meetings, we disseminated the PPEET and the newly developed patient‐reported clinical decision‐making and self‐management tool to patients who participated in the partnership meetings through online self‐administered questionnaires. The PPEET is designed to explore existing enablers and barriers related to patient engagement processes, as well as the impacts and influences of patient engagement [36]. The questionnaire contains 16 questions/statements in four categories: (i) communication and support for participation; (ii) sharing views and perspectives; (iii) impacts and influence of the engagement initiative; and (iv) final thoughts [37]. Three open‐ended questions were modified by our research team into multiple‐choice questions as per feedback from our PP, who reviewed and tested the questionnaire before deployment. The patient‐reported questionnaire comprises an 11‐item Likert scale for clinical decision‐making and a separate 11‐item Likert scale for self‐management. Questions/statements were rated using a 5‐point Likert scale, ranging from ‘strongly disagree’ to ‘strongly agree’.

Patients and research experts checked the questionnaires for their content validity, length appropriateness and questioning flow. We measured Cronbach's *α* coefficient for internal consistency reliability of the three Likert scales. Cronbach's *α* coefficient for the translated modified 11‐item Likert scale in PPEET was 0.9075, indicating excellent reliability. The clinical decision‐making 11‐item Likert scale in the patient‐reported questionnaire had an excellent level of reliability, as indicated by its Cronbach's α coefficient of 0.902. Also, Cronbach's *α* coefficient for the developed self‐management 11‐item Likert scale in the patient‐reported questionnaire was 0.75, indicating good reliability [38]. The language in all questionnaires was clear and comprehensible (File S3).

### 2.6 Data Analysis

Data were entered and analysed using the IBM SPSS statistics software (IBM SPSS Statistics for Windows, Version 26.0. Armonk, NY: IBM Corp.). The primary outcomes were distress levels and partners' experience of partnership. The secondary outcomes were the clinical decision‐making preferences and self‐management practices. For categorical variables, data were described as frequency and percentage [*n* (%)]. Mean with standard deviation (SD) was used to report continuous variables [mean (±SD)]. We used the Kolmogorov–Smirnov test to assess normality. We compared partners' demographics, personal characteristics, disease and therapeutic characteristics to primary and secondary outcomes. A paired *t*‐test was used to compare distress levels pre‐ and post‐partnership. ANOVA and Independent *t*‐tests were used to compare continuous variables. Categorical variables were analysed using the *χ* ^2^ test and Fisher's exact test. *p* values were two‐sided, with statistical significance set at *p*‐value < 0.05.

## 3 Results

### 3.1 Sample Characteristics

Of the 100 engaged participants, the majority of PP were patients (67%) and family members (33%). Most of the PP were female (63%) and male (37%) in the age group between 35 and 65 years old (68%). The majority of PP had an education level of less than a high school degree (35%), were not related to the healthcare industry (77%) and unemployed (57%). Further personal, disease and therapeutic characteristics are described in Table 1.

**Table 1:** Sample characteristics of patient partners and partnership experience.

	PP experience		
Patient partners characteristics	*n* (%)	*M* (SD)	*p*
PP Role^a^			0.8
Patient	67 (67%)	4 (0.5)	
Family member	33 (33%)	4 (0.4)	
Gender^a^			0.3
Female	63 (63%)	4.1 (0.4)	
Male	37 (37%)	3.9 (0.5)	
Age group^b^			0.2
18–25	1 (1%)	4.3	
>25–35	5 (5%)	4.3 (0.4)	
>35–65	68 (68%)	4 (0.5)	
>65	26 (26%)	4.1 (0.4)	
Level of education^b^			0.1
Unable to read and write	11 (11%)	3.8 (0.5)	
Less than high school	35 (35%)	4 (0.4)	
High school diploma	13 (13%)	3.9 (0.2)	
University degree	27 (27%)	4 (0.4)	
Post graduate/graduate degree	2 (2%)	4.1 (0.5)	
I prefer not to answer	12 (12%)	3.9 (0.4)	
Belong to health profession^a^			0.3
Yes	23 (23%)	4.1 (0.4)	
No	77 (77%)	4 (0.5)	
Employment status^b^			0.1
Employed full‐time	16 (16%)	4.2 (0.6)	
Employed part‐time	12 (12%)	4 (0.5)	
Self‐employed	7 (7%)	3.7 (0.6)	
Student	1 (1%)	4.3	
Retired	4 (4%)	4.3 (0.5)	
Unemployed	57 (57%)	4 (0.4)	
I prefer not to answer	3 (3%)	3.9 (0.4)	
Cancer diagnosis^b^			0.9
Haematologic	10 (10%)	4.2 (0.3)	
Lung cancer	10 (10%)	4 (0.4)	
Breast cancer	27 (27%)	4 (0.4)	
Gastrointestinal malignancy	18 (18%)	4 (0.6)	
Gynaecological malignancy	11 (11%)	4 (0.5)	
Others	24 (24%)	3.9 (0.4)	

Abbreviations: M, mean; n, number; *p*, *p*‐value; PP, patient partner; SD, Standard Deviation.

^a^ Independent *t*‐test conducted to compare PP Experience Index means among binary categorical variables.

^b^ One‐way ANOVA conducted to compare PP Experience Index means among variables with more than two categories.

### 3.2 Partners' Characteristics and Partnership

Patient Partnership Experience Mean Index was not statistically significantly different among patient characteristics variables, including gender, age groups, education level, employment level and cancer type.

### 3.3 Patient Engagement Evaluation

In the section on communication and supports for participation, PP mostly ‘agreed’ to ‘strongly agreed’ (4.4 ± 0.6) (86% response frequency) with (i) having a clear understanding of the purpose of the Partnership Committee; (ii) having the supports needed to participate; (iii) having enough information to contribute to the topics being discussed.

In sharing views and perspectives, PP mostly ‘agreed’ to ‘strongly agreed’ (4.2 ± 0.5) (95% response frequency) with (i) the ability to express views freely; (ii) felt that views were heard by the team.

In impacts and influence of the engagement initiative, PP mostly ‘agreed’ to ‘strongly agreed’ (3.6 ± 0.6) (63% response frequency) with (i) that Partnership Committee Meetings achieved its objectives; (ii) having the confidence that the input provided through this initiative will be used by SGH; (iii) the input provided through this activity will make a difference to the work of the organization; out of 100 PP, the influence of partnership committee reported were improved hospitalization experience and patient satisfaction (54%), respect and care provided by therapeutic communication (28%), shared decision making (26%) and other (9%).

In final thoughts, PP mostly ‘agreed’ to ‘strongly agreed’ (3.9 ± 0.5) (85.6% response frequency) with (i) becoming better informed about the quality of care, healthcare system management, care planning and evaluation; (ii) satisfaction with this engagement initiative; (iii) engagement initiative was a good use of the time (Figure S1).

### 3.4 Patient‐Reported Clinical Outcomes

More than half of PP reported experiencing side effects (54%). Out of 100 PP, the number of hospitalization due to complications was one time (8%), two times (4%), more than two times (8%) and never (84%).

### 3.5 Clinical Decision‐Making

In clinical communication, PP mostly ‘agreed’ to ‘strongly agreed’ (4 ± 0.5) (85% response frequency) with (i) Therapeutic Communication present; (ii) My viewpoints were heard during the treatment process; (iii) Healthcare provider took my contributions to the treatment.

In patient role in clinical decision‐making, PP mostly ‘agreed’ to ‘strongly agreed’ (3.8 ± 0.5) (73% response frequency) with (i) My input will influence final decisions related to the treatment; (ii) My family representatives and/or I had an equal opportunity with physician and other healthcare professionals to participate in clinical decision‐making; (iii) I clearly understand my role in the treatment process.

In inpatient education, PP mostly ‘agreed’ to ‘strongly agreed’ (3.7 ± 0.6) (65.3% response frequency) with (i) The information was available and easy to understand for me before or during the treatment process to participate knowledgeably; (ii) I was satisfied with the information given by the physician before, after or by the time of the doctor–patient discussion; (iii) I clearly understand what the treatment goals were.

In the therapeutic endpoint, PP mostly ‘agreed’ to ‘strongly agreed’ (4 ± 0.7) (79.5% response frequency) with (i) I would follow health advice from the healthcare professionals; (ii) I would recommend others to follow health advice from the healthcare professionals. Out of 100 PP, therapeutic clinical decision‐making preference was shared decision between patient and physician (84%), the decision by the physician alone (15%), the decision by the patient alone or the family in case the patient cannot (1%).

### 3.6 Self‐Management Practices

The mean of self‐management practice frequencies was (2.7 ± 0.4). In Medicine and Life Style (2.6 ± 0.5), PP mostly reported ‘All of the Time’ (66% response frequency) to (i) Monitor symptoms, bodily changes, treatment effects and/or disease risks and complications; ‘Most of the Time’ (41% response frequency) to (ii) Adjust your nutrition and diet; ‘Never’ (45% response frequency) to (iii) Adjust your exercise.

In Social support (2.7 ± 0.5), PP mostly reported ‘Most of the Time’ (38.5% response frequency) to (i) Seek support from relatives and friends; and (ii) Seek Support from healthcare professionals; ‘Some of the Time’ (38.6% response frequency) to (iii) Seek support from other cancer patients; engaging in (or online) support groups; (iv) Provide and/or arrange and/or join social support to friends and relatives; and (v) Limit social interactions to certain people or moments.

In Knowledge and information (2.9 ± 0.7), PP mostly reported ‘Some of the Time’ (37% response frequency) to (i) Seek information about disease and/or treatments; ‘Most of the Time’ (41% response frequency) to (ii) Seek information about self‐care; ‘Never’ (68% response frequency) to (iii) Avoid or neglect information.

### 3.7 Distress Level Pre‐ and Post‐Partnership

Paired T‐test determined that pre and post‐partnership distress levels were strongly and positively correlated (*r* = 0.78, *p* < 0.0001). There was a significant average difference between distress levels (*t* = 12.57, *p* < 0.0001). On average, before partnership distress levels (6.9 ± 2.8) were 2.3 points higher than after partnership distress levels (4.6 ± 2.7) (95% CI [1.93–2.65]). Also, the mean distress level for ‘disagree’ to partnership satisfaction (5.3 ± 3.1) was higher than the mean distress level for ‘strongly agree’ to partnership satisfaction (2.4 ± 2.5), *F*(3, 96) = 3.564, *p* = 0.017 (Figure S2).

### 3.8 Patient Partnership and Shared Clinical Decision‐Making

There was a statistically significant association between the Level of agreement with partnership satisfaction and therapeutic clinical decision‐making preference, *χ* ^2^(2) = 13.81, *p* = 0.025. Also, the partnership experience mean index was statistically significantly different among the therapeutic clinical decision‐making preference groups, mean partnership experience index for ‘shared decision between patient and physician’ (4.1 ± 0.4) was the highest, *F*(2, 97) = 3.419, *p* = 0.037.

The relationship between the clinical decision‐making mean index and self‐management practices mean index was positive, weak in strength and statistically significant; *r*(98) = 0.3, *p* = 0.004. The mean clinical decision‐making process index for ‘none’ hospitalization due to complication (4 ± 0.4) was higher than the mean clinical decision‐making process index of ‘two times’ hospitalization due to complication (3.2 ± 0.5), *F*(3, 96) = 4.269, *p* = 0.007.

### 3.9 Patient Partnership and Disease Self‐Management Practices

The mean of self‐management practices frequencies was statistically significantly different between satisfaction with partnership experience groups; mean self‐management practices for ‘disagree’ (2.4 ± 0.1) was lower than mean self‐management practices of ‘strongly agree’ (2.8 ± 0.3), *F*(3, 11.87) = 7.294, *p* = 0.005. Our study findings based on analytical statistics are grouped in Table 2.

**Table 2:** Analytical statistical results of partnership impact on distress level, therapeutic clinical decision‐making preference and self‐management practices.

	Mean (SD), *n* = 100				
Variable	Pre	Post	Mean difference (95% CI)	*t*‐statistic (*df*)	*p*
Distress level	6.9 (2.8)	4.6 (2.7)	95% CI [1.93–2.65]	12.57 (99)	<**0.0001** a

Satisfaction with partnership experience	*n*	After the partnership distress level, mean (SD)	*F*‐statistic (*df*1, *df*2)^b^	*p*
Disagree	3	5.3 (3.1)	3.564 (3,96)	**0.017** ^c^
Neither agree nor disagree	10	3.6 (2.6)		
Agree	77	5 (2.6)		
Strongly agree	10	2.4 (2.5)		

Therapeutic clinical decision‐making preference	*n*	Partnership experience index, mean (SD)	*F*‐statistic (*df*1, *df*2)^b^	*p*
The decision by the patient alone or the family in case the patient cannot	1	3.6	3.419 (2, 97)^d^	**0.037** d
Shared decision between patient and physician	84	4.1 (0.4)		
The decision by the physician alone	15	3.8 (0.5)		

Number of hospitalizations due to complication	*n*	Clinical decision‐making process index, mean (SD)	*F*‐statistic (*df*1, *df*2)^b^	*p*
One	8	4.1 (0.6)	4.269 (3, 96)	**0.007** e
Two	4	3.2 (0.5)		
More than two	8	4 (0.4)		
None	80	3.9 (0.4)		

Satisfaction with partnership experience	*n*	Self‐management practices frequencies, mean (SD)	*F*‐statistic (*df*1, *df*2)^f^	*P*
Disagree	3	2.4 (0.1)	7.294 (3, 11.87)	**0.005** g
Neither agree nor disagree	10	2.7 (0.3)		
Agree	77	2.8 (0.4)		
Strongly agree	10	2.8 (0.3)		

	Therapeutic clinical decision‐making preference, *n* (%)					
Satisfaction with partnership experience	Decision by the patient alone or the family in case the patient cannot	Shared Decision between patient and physician	The decision by the physician alone	*n*	*χ* ^2^‐statistics (*df*)^h^	*p*
Disagree	0	3 (100%)	0	3	13.81 (2)	**0.025** i
Neither agree nor disagree	1 (10%)	5 (50%)	4 (40%)	10		
Agree	0	66 (85.7%)	11 (14.3%)	77		
Strongly agree	0	10 (100%)	0	10		

	Clinical decision‐making mean index	Self‐management practices mean index
Clinical decision‐making mean index	—	
Self‐management practices mean index	**0.3** j	—

^a^ Paired *t*‐test.

^b^ One‐way ANOVA.

^c^ Post hoc analysis with Games–Howell shows a significant difference between Agree and Strongly Agree (*p* = 0.04).

^d^ Post hoc analysis could not be performed.

^e^ Post hoc analysis showed no significance.

^f^ Welch's ANOVA.

^g^ Post hoc analysis with Games‐Howell shows a significant difference between Agree and Disagree (*p* = 0.02) and between Strongly Agree and Disagree (*p* = 0.02).

^h^ Fisher's exact test.

^i^ 8 cells (66.7%) have expected count less than 5. The minimum expected count is 0.03.

^j^ Pearson's correlation coefficient (*r*), *r*[98] = 0.3, *p* = 0.004.

## 4 Discussion

### 4.1 Partner Characteristics and Impact on Partnership Experience

Patient partnership programs yield beneficial outcomes for patients, healthcare professionals and organizations, although they adopted unconventional approaches in various studies. In this study, we aim to evaluate the cancer patients' experience as partners in the quality improvement committee. Unlike previous studies, we investigated the impact of the partnership model of care on therapeutic clinical decision‐making preferences, self‐management practices and cancer‐related distress.

Compared with other studies of similar quasi‐experimental methodology, our PP recruited were higher in number [14, 16, 17, 18], but of similar characteristics. The majority of PP were patients unlike in Dayekh et al, where family members were dominant as PP [14]. Similar to most of the studies, we observed large participation from women PP [17, 18, 19, 20, 21, 22, 39]. None of the studies restricted partners' selection to a specific age group, as in our research, most of the participants belonged to the age category between 35 and 65 years old [14, 16, 17, 18, 19, 21, 22, 23, 24, 25, 40, 41, 42, 43, 44, 45]. Unlike other studies that had different partners with chronic disease experiences, we only recruited cancer patients as partners. Our PP dealt with care mainly related to Breast cancer diagnosis, similar to Brouwers et al. [18] and most of the PP were in their Late III stage as in the Ohle ´n et al. study [43]. Similar to Inan et al., our sample's therapeutic plan was majorly chemotherapy [42]. Nearly all PP did not have previous partnership experience, unlike half of the Omeni et al. study [26].

Our study sample resembled the literature in representing mainly the disadvantaged patient population characterized by low education and income levels. This study included majorly PP with a high school degree, similar to most of the studies [17, 18, 22, 42, 43]. Samudre et al. recruited PP who had less than seven‐grade education levels [25]. Whereas, other studies had most of the PP with university graduate degrees [20, 27, 41]. Also, PP did not need to belong to the healthcare industry to be engaged reinforced by two previous partnership publications [14, 17]. More than half of the PP were unemployed in low‐income situations similar to Inan et al. [42].

Although all of the previous partnership articles described the engagement of partners of diverse ages, genders, races, education levels and degrees of experience interacting with healthcare professionals, none of them reported a statistical association between the partners' characteristics and partnership [14, 16, 17, 18, 19, 20, 21, 22, 23, 24, 25, 26, 27, 28, 29, 30, 31]. We were able to statistically prove that partnership experience was not related to the partners' characteristics, most importantly neither to education level nor employment status.

### 4.2 Partnership Experience Evaluation

Similar to previous results, participants in this study reported ‘agreed’ to ‘strongly agreed’ on all four components of the PPEET, which are communication and support for participation, sharing views and perspectives, impacts and influence of the engagement initiative and satisfaction with partnership experience [19, 27, 46]. Our study findings matched most of the previous studies in context related to communication and team support. Most studies reported that participation was valued by users [16, 20, 28, 46], as they felt enthusiastic and needed [20]. Similarly, our participants were satisfied with the engagement initiative [14, 46]. However, less than half of the partners in the Abelson et al. study reported that they thought about quitting [20].

We found partnership benefits similar to those reported in the literature. Our participants considered improved hospitalization experience and patient satisfaction as the advantage of the partnership. Similar to previous studies, they found that partnership is essential to shaping the healthcare system to more patient‐centred oriented care [14, 16, 20, 22, 23, 26, 27, 28, 46, 47]. Studies by Dayekh et al. and Galvin et al. mentioned the patients' ability to express their opinions through engagement programs, similar to our study [14, 16]. Only Dayekh et al and our study reported hospital reputation enhancement and patient loyalty as partnership advantages [14].

Participants in this study recommended the need to have educational materials provided for partners. Similar to the Brown et al study, partners suggested training before engagement [48]. Our participants reported the need for discussion guidance during meetings to better manage team dynamics and communication, similar to some of the studies [14, 17, 27]. As in almost all studies, we found time and availability to attend meetings as a challenge [14, 16, 17, 18, 27, 31, 48]. Half of our participants reported nothing for improvement and no challenges faced, similar to Dayekh et al. [14]. However, different areas for improvement were only mentioned in previous literature; (i) engaging patients earlier in planning; [23, 27, 30] (ii) sufficient partner representation; [20, 25, 27, 49] (iii) physical environment; [50] (iv) financial cost; [18] (v) language and medical terminology [17, 31].

### 4.3 Distress Level Pre‐ and Post‐Partnership

PP engaged in the co‐design of the healthcare system in this study mostly reported patient distress relief as an advantage. Equivalent to Brown et al. study, indicating that partnership participation was a source of positivity to partners [48]. In contrast, Omeni et al., found that partnership can negatively impact the health and self‐esteem of participants due to induced stress [26].

We assessed for the first time the impact of the partnership initiative on the cancer patient distress level, unlike current published research. We proved that pre‐partnership distress levels (6.9 ± 2.8) were 2.3 points higher than post‐partnership distress levels (4.6 ± 2.7).

### 4.4 Partnership and Clinical Decision‐Making Preference

A strong point of this study is that it evaluates the partnership model of care's effect on clinical decision‐making and self‐management practices of cancer patients. Our study emphasizes that the partnership model had a statistically significant association with therapeutic clinical decision‐making preference. Analogously, after providing thorough chemotherapy consulting services to cancer patients, a study discovered a significant increase in clinical co‐decision‐making scores [45]. We demonstrated that the patients with higher clinical decision‐making scores had less hospitalization due to complications. Another cross‐sectional qualitative study, found homogenous results, indicating that engaging patients in empowerment activities, such as taking part in advocacy and support groups and shared decision‐making, may promote health literacy and help to improve patient outcomes [39].

The majority of participants in this partnership study chose shared decisions between patient and physician as therapeutic clinical decision‐making preference. Unlike Gu et al. study, where none of the patients chose shared decision‐making between patients and physicians. However, we had common responses to clinical decision‐making preferences, which were decisions by the physician alone and decisions by the patient alone or by family in case the patient cannot [44].

### 4.5 Partnership Impact on Self‐Management Practices

In parallel with previous results, we found a positive and statistically significant correlation between clinical decision‐making scores and self‐management scores [47, 51]. We demonstrated that patient partnership significantly improves disease self‐management in accordance with current literature [52, 53, 54]. In addition, Dongen et al. study recommended implementing patient–professional partnerships to maximize the effectiveness of self‐management, taking into consideration that cancer patients with more depressive symptoms and lower levels of physical functioning education might have more difficulties with certain self‐management strategies [55].

### 4.6 Strength and Limitations

Strengths of this research included the use of robust quantitative methods for ensuring rigour reporting of the first assessment of distress level post‐partnership intervention and the impact of the partnership model of care on the clinical decision‐making and self‐management practices for cancer patients. The research was conducted by an interdisciplinary research team experienced in both clinical research and healthcare system quality improvement, and it included PP with hospital experience. Even though partnership with patients is a relatively new practice, the sampling strategy was random without restriction to choosing participants who were trained and had high education levels.

Certain drawbacks to this study warranted discussion. All participants were recruited from one hospital; thus, findings may not be relevant to hospitals with differing health systems. The sole data source was the subjective assessment provided by PP; daily engagement and observation with patients and their healthcare providers would have provided complementary objective data gathering. Additionally, a monitoring system based on carefully chosen strategic key performance indicators is required for such experimental research to obtain substantial proof of the superiority of the partnership model of care.

## 5 Conclusion

Cancer patients face a serious problem of non‐adherence to treatment, which increases complications from disease, risk of death and medical costs. Consequently, policymakers have been compelled to develop ways to improve clinical outcomes and reduce complications by optimizing disease self‐management techniques. Thus, more patient involvement in healthcare decision‐making is consistent with optimizing the quality of healthcare and patient's clinical outcomes. In addition to clinical consulting positions, healthcare system design, implementation and evaluation are examples of strategies to engage patients. This study showed that the partnership model of care produced favourable outcomes. Despite diverse ages, gender, education levels, employment status and disease characteristics, cancer patients as partners had optimal engagement experience in quality improvement projects. Participants were satisfied with this engagement initiative and found it a good use of their time. The main partnership benefit was improved hospitalization experience. Recommendations for improvement were training, team dynamics management and proper time allocation. Distress level post the implementation of the partnership approach was significantly reduced. This study highlights the importance of patient partnership and ‎its ability to influence shared decision‐making and self‐management practices, especially for cancer patients. Healthcare professionals should engage patients in clinical decision‐making processes for better clinical outcomes. We recommend medical staff tailor the diverse patient engagement tools based on the patient's preferences and tolerance for optimal empowerment. All patients must have equal opportunities for engagement in healthcare system design after receiving sufficient education. This will preserve favourable patient clinical outcomes in parallel with sustainable healthcare system transformation and organizational development. Further research is needed to monitor healthcare institution growth after embedding a partnership model of care into the system and organizational culture.

## Author Contributions

**Zahraa Alrayshouni:** conceptualization, investigation, methodology, visualization, resources, writing–original draft, writing–review and editing. **Alaa Dayekh:** conceptualization, methodology, software, validation, formal analysis, supervision, visualization, project administration, resources, writing–original draft, writing–review and editing, data curation. **Ahmad El‐Tassi:** supervision, project administration, resources, writing–review and editing, validation. **Annamária Pakai:** validation, project administration, supervision, writing–review and editing.

## Ethics Statement

We further confirm that any aspect of the work covered in this manuscript that has involved human patients has been conducted with the ethical approval of all relevant bodies and that such approvals are acknowledged within the manuscript. An IRB approval was obtained. The IRB ID number is 2023‐H‐168‐HS‐M‐0573.

## Consent

Written consent to participate in the patient partnership committee for quality improvement was obtained from the patient(s) or their legal guardian(s).

## Conflicts of Interest

The authors declare no conflicts of interest.

## Supporting information

FIGURE S1 Partnership experience level of agreement. PP considered improved hospitalization experience and patient satisfaction (49%), patient's ability to express their opinion (39%), patient distress relief (21%), hospital reputation enhancement and patient loyalty (21%) and other (5%) were partnership committee strengths and advantages. Moreover, PP expressed the need to have educational materials provided for patient partners (19%), discussion guidance during meetings to avoid conflicts (12%), time and availability to attend meetings (7%) and other diverse recommendations (14%). While almost half of PP reported nothing for improvement and no challenges faced (49%).

FIGURE S2 Pre‐ and post‐partnership distress levels.

FILE S3 Study questionnaires.

## Data Availability

The data that support the findings of this study are openly available in figshare at https://figshare.com/s/09b83d694b41827a0e5e, reference number https://doi.org/10.6084/m9.figshare.25015778.
