# Supplementary figures and images for: Rationalizing the Influence of Co‐Design on Distress, Clinical Decision‐Making and Disease Self‐Management of Cancer Patients‐as‐Partners: A Quasi‐Experimental Study
Source: Health Expect. 2024 Jun 14;27(3):e14113. doi: 10.1111/hex.14113 (PMC11176735; doi:10.1111/hex.14113)

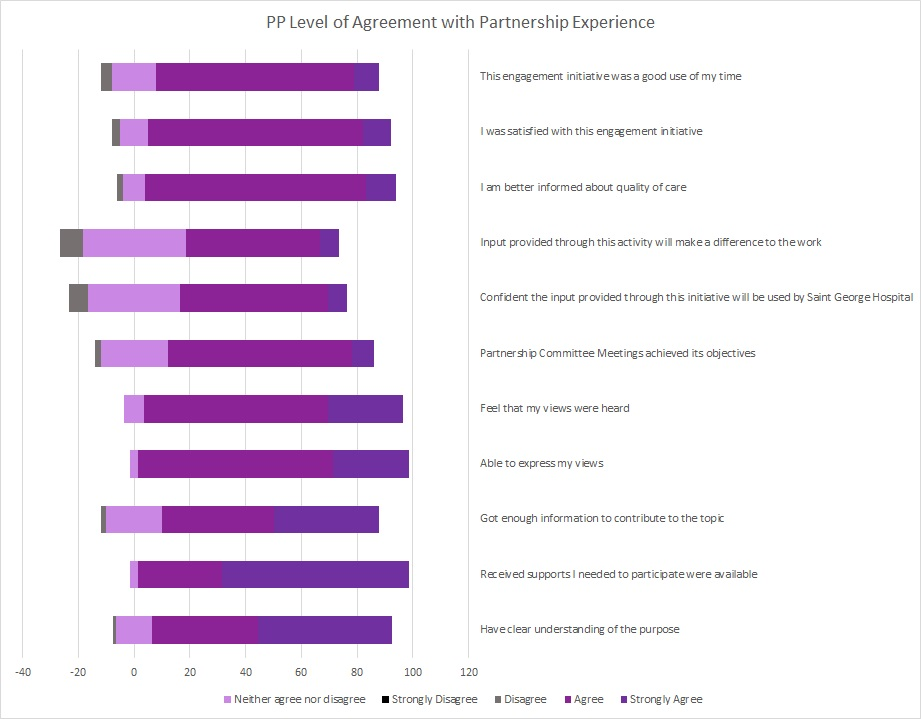

Supplement: Supplementary file 1 — FIGURE S1 Partnership experience level of agreement. PP considered improved hospitalization experience and patient satisfaction (49%), patient's ability to express their opinion (39%), patient distress relief (21%), hospital reputation enhancement and patient loyalty (21%) and other (5%) were partnership committee strengths and advantages. Moreover, PP expressed the need to have educational materials provided for patient partners (19%), discussion guidance during meetings to avoid conflicts (12%), time and availability to attend meetings (7%) and other diverse recommendations (14%). While almost half of PP reported nothing for improvement and no challenges faced (49%). [file HEX-27-e14113-s002.tiff]

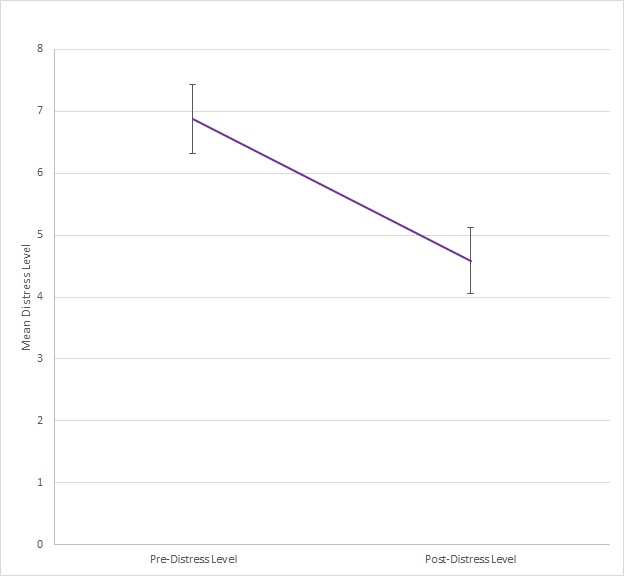

Supplement: Supplementary file 2 — FIGURE S2 Pre‐ and post‐partnership distress levels. [file HEX-27-e14113-s003.tiff]
