# Supplementary material for: Rationalizing the Influence of Co‐Design on Distress, Clinical Decision‐Making and Disease Self‐Management of Cancer Patients‐as‐Partners: A Quasi‐Experimental Study
Source: Health Expect. 2024 Jun 14;27(3):e14113. doi: 10.1111/hex.14113 (PMC11176735; doi:10.1111/hex.14113)
Supplement: Supplementary file 3 — FILE S3 Study questionnaires. [file HEX-27-e14113-s001.docx]

**Patient Partner Characteristics Survey**

The purpose of collecting this information is to gain a deeper understanding of your background so we can understand how well we have done in engaging relevant individuals and communities in our engagement initiatives. Please note that all responses are confidential, and we value all individuals’ contributions to this work. You may skip any question you would prefer not to answer.

1. Name:
2. Gender: □ Male □ Female
3. Age:

□ 18 - 25

□ >25 - 35

□ >35 - 65

□ >65

1. Marital Status:

□ Married

□ Separated

□ Single

□ Widowed

1. What is the highest level of education that you have completed? *Please select one.*

| □  □ | Unable to Read and Write  Less than high school |
| --- | --- |
| □ | High school diploma |
| □ | University Degree |
| □ | Post graduate, professional or graduate degree |
| □ | I prefer not to answer |

1. Have you ever worked in a Health Profession? □ Yes □ No
2. What is your current employment status? *Please select all that apply.*

| □ | Employed full-time |
| --- | --- |
| □ | Employed part-time |
| □ | Self-employed |
| □ | Student |
| □ | Retired |
| □ | Unemployed |
| □ | I prefer not to answer |

1. Governorates: □ Beirut □ Mount Lebanon □ North □ Beqaa □ Nabatiye □ South
2. Best day to contact you: □Monday □Tuesday □Wednesday □Thursday □Friday □Saturday □Sunday □Check All
3. How would you like to participate in the partnership model of care?

□In-person (such as meetings) □Phone □Skype □Other:

1. What are some issues that are of special interest to you?

□ Patient-Physician Communication

□ safety

□ Hospitalization experience and quality

□ Hospital logistics

□ Staff performance

□ others

1. Are you a patient with a cancer diagnosis, or a family member or representative to a patient with cancer? □Yes □ No
2. Do you consider yourself being in a period of stable health? □Yes □ No
3. Do you have a comprehensive analytical thinking? □ Yes □ No
4. Do you have good communication skills? □Yes □ No
5. Have you previously participated in any partnership model of care initiative?

**Disease characteristics**

1. Smoking Status: □Yes □ No
2. Family and Social Support: □Yes □ No
3. Travel distance to hospital: □< 30 min □30-60 min □1-3 hours □3-6 hours □> 6 hours
4. Family history of cancer: □ Yes □ No
5. Type of cancer:

□ Hematologic (Non-Hodgkin Lymphoma and Hodgkin Lymphoma)

□ Lung cancer

□ Breast cancer

□ Gastrointestinal malignancy

□ Gynecological malignancy

□ Others:

1. Stage of cancer:

□ Early I

□ Early II

□ Late III

□ Late IV

1. History of co-morbidity: □ Yes □ No
2. Is the patient a recurrent cancer patient? □ Yes □ No

**Therapeutic Plan**

1. Treatment modality

| □ | Chemotherapy only |
| --- | --- |
| □ | Chemotherapy and surgery |
| □ | Radiotherapy Only |
| □ | Radiotherapy, Chemotherapy and surgery |
| □ | Radiotherapy and Surgery |

1. Number of chemotherapies: □ Mono chemotherapy □ Poly chemotherapy
2. Number of chemotherapy cycles:

□ 1st cycle

□ 2nd cycle

□ 3rd cycle

□ Greater than three

1. Side effect: □ Yes □ No
2. Number of hospitalizations due to complications:

□ One

□ Two

□ More than two

□ None

1. Type of payment:

□ MOPH

□ Full coverage

□ self-pay

1. □ Insurance

□Yes □No

**Patient Partnership Experience Questionnaire**

“The Public and Patient Engagement Evaluation Tool has been licensed under a Creative Commons Attribution-Non Commercial-Share Alike 4.0 International License. ©2018, Julia Abelson and the PPEET Research-Practice Collaborative. McMaster University. All rights reserved.”

**Participants’ Engagement Activities Questionnaire**

We are interested in your feedback about your participation in patient partnership meetings.

The questionnaire includes 16 statements or questions – some questions ask you to rate your level of agreement in response to a statement and others ask for you to provide your comments and feedback. You are encouraged to share your experiences and any information you feel is relevant and will help us to improve our engagement processes.

We appreciate your feedback. It is critical to helping us improve our engagement processes. Please be aware that all of the information you provide will be confidential. Thank you for your participation!
Background Information

1. What perspective did you bring to Committee Meetings?

| 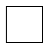 | Patient/patient advisor/patient partner |
| --- | --- |
| 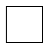 | Family member/caregiver |
| 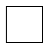 | Staff member |
| 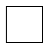 | Others, please specify: |

1. How long have you been working with Hospital as an employee or patient/family/community partner?

| 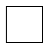 | This is my first participation |
| --- | --- |
| 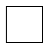 | Less than 6 months |
| 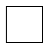 | 6 – 12 months |
| 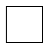 | 1 – 2 years |

Part A. Communication and Supports for Participation

Please indicate your level of agreement with each of the statements below.

1. I had a clear understanding of the purpose of Partnership Committee Meetings.

| 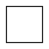 | Strongly disagree | 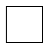 | Disagree | 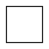 | Neither agree nor disagree | 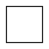 | Agree | 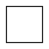 | Strongly agree |
| --- | --- | --- | --- | --- | --- | --- | --- | --- | --- |

1. The supports I needed to participate were available (e.g., transportation, guidance, etc.)

| 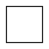 | Strongly disagree | 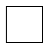 | Disagree | 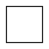 | Neither agree nor disagree | 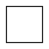 | Agree | 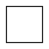 | Strongly agree |
| --- | --- | --- | --- | --- | --- | --- | --- | --- | --- |

1. I had enough information to contribute to the topic being discussed.

| 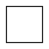 | Strongly disagree | 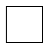 | Disagree | 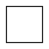 | Neither agree nor disagree | 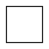 | Agree | 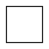 | Strongly agree |
| --- | --- | --- | --- | --- | --- | --- | --- | --- | --- |

Part B. Sharing Your Views and Perspectives

1. I was able to express my views freely.

| 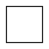 | Strongly disagree | 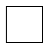 | Disagree | 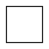 | Neither agree nor disagree | 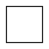 | Agree | 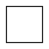 | Strongly agree |
| --- | --- | --- | --- | --- | --- | --- | --- | --- | --- |

1. I feel that my views were heard.

| 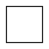 | Strongly disagree | 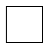 | Disagree | 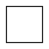 | Neither agree nor disagree | 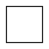 | Agree | 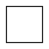 | Strongly agree |
| --- | --- | --- | --- | --- | --- | --- | --- | --- | --- |

Part C. Impacts and Influence of the Engagement Initiative

1. I think that Partnership Committee Meetings achieved its objectives.

| 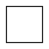 | Strongly disagree | 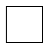 | Disagree | 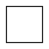 | Neither agree nor disagree | 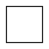 | Agree | 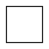 | Strongly agree |
| --- | --- | --- | --- | --- | --- | --- | --- | --- | --- |

1. I am confident the input provided through this initiative will be used by the Hospital

| 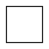 | Strongly disagree | 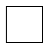 | Disagree | 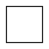 | Neither agree nor disagree | 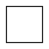 | Agree | 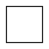 | Strongly agree |
| --- | --- | --- | --- | --- | --- | --- | --- | --- | --- |

1. I think the input provided through this activity will make a difference to the work of the organization.

| 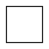 | Strongly disagree | 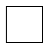 | Disagree | 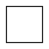 | Neither agree nor disagree | 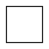 | Agree | 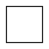 | Strongly agree |
| --- | --- | --- | --- | --- | --- | --- | --- | --- | --- |

1. What else would you like us to know about the influence you think Partnership Committee Meetings will have?

□ Respect and Care provided by therapeutic communication

□ Improved hospitalization experience and patient satisfaction

□ shared decision making

□ Other

Part D. Final Thoughts

1. As a result of my participation in Partnership Committee Meetings, I am better informed about quality of care, healthcare system management, care planning and evaluation…

| 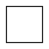 | Strongly disagree | 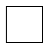 | Disagree | 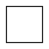 | Neither agree nor disagree | 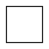 | Agree | 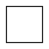 | Strongly agree |
| --- | --- | --- | --- | --- | --- | --- | --- | --- | --- |

1. Overall, I was satisfied with this engagement initiative.

| 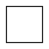 | Strongly disagree | 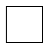 | Disagree | 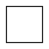 | Neither agree nor disagree | 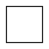 | Agree | 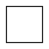 | Strongly agree |
| --- | --- | --- | --- | --- | --- | --- | --- | --- | --- |

1. This engagement initiative was a good use of my time.

| 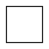 | Strongly disagree | 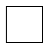 | Disagree | 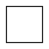 | Neither agree nor disagree | 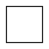 | Agree | 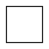 | Strongly agree |
| --- | --- | --- | --- | --- | --- | --- | --- | --- | --- |

1. What were the strengths/advantages of Partnership Committee Meetings?

□ patient distress relief

□ improve hospitalization experience and satisfaction

□ patient express opinion

□ hospital reputation enhancement and patient loyalty

□ others

1. What could be improved about Partnership Committee Meetings?

□ nothing for improvement, no challenges faced

□ time and availability to attend meetings

□ discussion guidance to avoid conflicts

□ educational materials provided for patients

□ others

Thank you for your participation.

**Patient-Reported Clinical decision making and Self-Management Survey**

The purpose of collecting this information is to gain a deeper understanding of your background so we can understand how well the healthcare professionals have done in engaging patients and relevant individuals in treatment. Please note that all responses are confidential, and we value all individuals’ contributions to this work. You may skip any question you would prefer not to answer.

1. Name:

**Health-care system (Degree of involvement in clinical decision making)**

Please indicate your level of agreement with each of the statements below on a scale from 5 till 1, where 5 is strongly agree and 1 strongly disagree.

1. I believe there was Therapeutic Communication present.

| 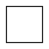 | Strongly disagree | 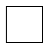 | Disagree | 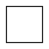 | Neutral | 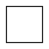 | Agree | 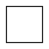 | Strongly agree |
| --- | --- | --- | --- | --- | --- | --- | --- | --- | --- |

1. I believe that my viewpoints were heard during the treatment process.

| 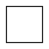 | Strongly disagree | 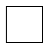 | Disagree | 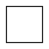 | Neutral | 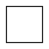 | Agree | 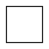 | Strongly agree |
| --- | --- | --- | --- | --- | --- | --- | --- | --- | --- |

1. The healthcare provider took my contributions to the treatment process seriously.

| 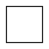 | Strongly disagree | 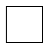 | Disagree | 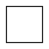 | Neutral | 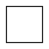 | Agree | 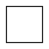 | Strongly agree |
| --- | --- | --- | --- | --- | --- | --- | --- | --- | --- |

1. I believe that my input will influence final decisions related to the treatment choices.

| 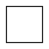 | Strongly disagree | 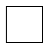 | Disagree | 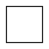 | Neutral | 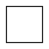 | Agree | 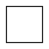 | Strongly agree |
| --- | --- | --- | --- | --- | --- | --- | --- | --- | --- |

1. My family representatives and/or I had equal opportunity with physician and other healthcare professionals to participate in clinical decision making.

| 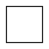 | Strongly disagree | 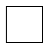 | Disagree | 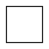 | Neutral | 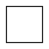 | Agree | 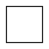 | Strongly agree |
| --- | --- | --- | --- | --- | --- | --- | --- | --- | --- |

1. I clearly understand my role in the treatment process.

| 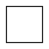 | Strongly disagree | 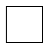 | Disagree | 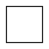 | Neutral | 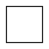 | Agree | 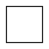 | Strongly agree |
| --- | --- | --- | --- | --- | --- | --- | --- | --- | --- |

1. The information was available easy to understand to me before or during the treatment process so as to participate knowledgeably.

| 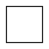 | Strongly disagree | 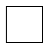 | Disagree | 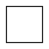 | Neutral | 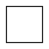 | Agree | 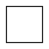 | Strongly agree |
| --- | --- | --- | --- | --- | --- | --- | --- | --- | --- |

1. I was satisfied with information given by the physician before, after or by the time of the doctor-patient discussion.

| 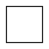 | Strongly disagree | 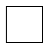 | Disagree |  | Neutral |  | Agree |  | Strongly agree |
| --- | --- | --- | --- | --- | --- | --- | --- | --- | --- |

1. I clearly understand what the treatment goals were.

|  | Strongly disagree |  | Disagree |  | Neutral |  | Agree |  | Strongly agree |
| --- | --- | --- | --- | --- | --- | --- | --- | --- | --- |

1. I would follow health advice from the healthcare professionals.

|  | Strongly disagree |  | Disagree |  | Neutral |  | Agree |  | Strongly agree |
| --- | --- | --- | --- | --- | --- | --- | --- | --- | --- |

1. I would recommend others to follow health advice from the healthcare professionals.

|  | Strongly disagree |  | Disagree |  | Neutral |  | Agree |  | Strongly agree |
| --- | --- | --- | --- | --- | --- | --- | --- | --- | --- |

1. In your opinion, who should participate in decision on therapy? (decision by patient alone, shared with the physician or by the physician alone)

|  | Decision by patient alone or family incase patient cannot |  | Decision shared with physician |  | Decision by physician alone |
| --- | --- | --- | --- | --- | --- |

**Self-management practices**

1. How often do you monitor symptoms, bodily changes, treatment effects and/or disease risks and complications?

|  | None of the time |  | Some of the time |  | Most of the time |  | All of the time |
| --- | --- | --- | --- | --- | --- | --- | --- |

1. How often do you adjust your nutrition and diet?

|  | None of the time |  | Some of the time |  | Most of the time |  | All of the time |
| --- | --- | --- | --- | --- | --- | --- | --- |

1. How often do you adjust your exercise (e.g. exercising more, balancing rest and physical activity)?

|  | None of the time |  | Some of the time |  | Most of the time |  | All of the time |
| --- | --- | --- | --- | --- | --- | --- | --- |

1. How often do you seek support from relatives and friends?

|  | None of the time |  | Some of the time |  | Most of the time |  | All of the time |
| --- | --- | --- | --- | --- | --- | --- | --- |

1. How often do you seek support from healthcare professionals?

|  | None of the time |  | Some of the time |  | Most of the time |  | All of the time |
| --- | --- | --- | --- | --- | --- | --- | --- |

1. How often do you seek support from other cancer patients; engaging in (or online) support groups?

|  | None of the time |  | Some of the time |  | Most of the time |  | All of the time |
| --- | --- | --- | --- | --- | --- | --- | --- |

1. How often do you provide and/or arrange and/or join social support to friends and relatives?

|  | None of the time |  | Some of the time |  | Most of the time |  | All of the time |
| --- | --- | --- | --- | --- | --- | --- | --- |

1. How often do you limit social interactions to certain people or moments (e.g. selective communication, social isolation)?

|  | None of the time |  | Some of the time |  | Most of the time |  | All of the time |
| --- | --- | --- | --- | --- | --- | --- | --- |

1. How often do you seek information about disease and/or treatments?

|  | None of the time |  | Some of the time |  | Most of the time |  | All of the time |
| --- | --- | --- | --- | --- | --- | --- | --- |

1. How often do you seek information about self-care?

|  | None of the time |  | Some of the time |  | Most of the time |  | All of the time |
| --- | --- | --- | --- | --- | --- | --- | --- |

1. How often do you avoid or neglect information?

|  | None of the time |  | Some of the time |  | Most of the time |  | All of the time |
| --- | --- | --- | --- | --- | --- | --- | --- |

**إستمارة الشريك من المرضى والعائلة**

إن الهدف من جمع المعلومات في هذه اللإستمارة هو إستحصال على قدر كاف من المعلومات التي تخولنا إختيار الشريك المناسب من المرضى والعائلة والمتقدمين للمشاركة بفعاليات الشراكة مع المريض. الرجاء أخذ العلم بأننا نحترم ونطبق أسس السريّة المهنيّة بحيث أن جميع المعلومات تخضع للسريّة التامة ولن يتم الإطلاع عليها إلا من قبل فريق العمل في المستشفى. نحن نقدر مشاركتكم الفعالة. يمكنكم عدم الإجابة عن أي سؤال بحسب تطلعاتكم.

1. الإسم:

2. الجنس: □ ذكر□ أنثى

3. العمر:

18 – 25 □

>25 – 35 □

>35 – 65 □

>65 □

4. الحالة الإجتماعيّة:

□ متزوج

□ منفصل

□ أعزب

□ أرمل

5. ما هو أعلى مستوى تعليمي قد حصلته؟ إختر إجابة واحدة.

□ لا أعرف القراءة ولا الكتابة

□ متوسط

□ ثانوي

□ إجازة جامعيّة

□ دراسات عليا

□ أفضل عدم الإجابة

6. هل تعمل حالياً أو عملت مسبقاً ضمن القطاع الصحي؟ □ كلا□ نعم

7. هل تعمل في الوقت الحالي؟ إختر كل ما هو مناسب.

□موظف بدوام كامل

□موظف بدوام جزئي

□أعمل لحسابي الشخصي

□طالب

□متقاعد

□لا أعمل حالياً

□أفضل عدم الإجابة

8. المحافظة: □ بيروت □ جبل لبنان □ الشمال □ البقاع □ النبطيّة □ الجنوب

9. إختر اليوم المناسب ليتم النواصل معك: □الإثنين □الثلاثاء □الأربعاء □الخميس □الجمعة □السبت □الأحد □جميع أيام الأسبوع

10. كيف تفضل المشاركة بفعاليات الشراكة مع المريض؟

□الحضور الشخصي (عبر الإجتماعات) □عبر الهاتف □عبر سكايب Skype □طرق أخرى تفضلها:

11. ما هي المواضيع التي يهمك مناقشتها خلال إجتماعات الشراكة مع المريض المتعلقة بتصميم, تنفيذ وتقييم إدارة أنظمة الرعاية الصحيّ ؟

□ التواصل ما بين الطبيب والمريض

□ السلامة

□ التجربة الإستشفائيّة وجودة الرعاية المقدمة

□ الأمور اللوجستيّة في المستشفى (الطعام, النظافة, هندسة البناء...)

□ آداء الفريق الطبي والإداري

□ غير ذلك

12. هل أنت مريض, أو فرد من العائلة, أو تهتم لمريض تم تشخيصه بمرض السرطان؟ □نعم □ كلا

13. . هل تعد نفسك بصحة جيدة أو بوضع صحي مستقر؟ □نعم □ كلا

14. هل لديك قدرة تحليليّة ونقد بنّاء؟ □نعم □ كلا

15. هل تمتلك مهارات التواصل الفعّالة؟ □نعم □ كلا

16. هل شاركت مسبقاً بفعاليات الشراكة مع المريض؟ □نعم □ كلا

**إستمارة تقييم فعاليات الشراكة مع المريض**

يهمنا تحصيل التغذية الراجعة لتقييم تجربة المشاركة كعضو فعال ضمن فعاليات لجنة الشراكة مع المريض. تتضمن هذه الإستمارة 16 بنداً سيتم الطلب منك إختيار الإجابة المناسبة أو الإبداء والتعبير عن رأيك لتحسين تجربة الشراكة. الرجاء الإنتباء الى أننا نحترم ونطبق مبادئ السريّة المهنيّة وأن جميع المعلومات سيتم الإطلاع عليها من قبل المعنيين في المستشفى بهدف التطوير من آليات العمل وتحسين تجربة الشراكة مع المريض.

شكراً لكم على المشاركة.
**معلومات عامة**

1. ما كان دورك ضمن فعاليات لجنة الشراكة مع المريض؟

|  | □ شريك من المرضى |
| --- | --- |
|  | □ فرد من عائلة, ممثل عن المريض |
|  | □ موظف في المستشفى |
|  | □ غير ذلك الرجاء التحديد |

1. حدد المدة الزمنيّة لمشاركتك ضمن إجتماعات لجنة الشراكة مع المريض.

|  | □ هذه أول مشاركة |
| --- | --- |
|  | □ أقل من ستة أشهر |
|  | □ 6 – 12 شهراً |
|  | □ سنة الى سنتين |

التواصل ودعم المشاركة

حدد الى أي مدى أنت موافق على الإجابات أدناه.

1. لديّ فهم واضح لأهداف إجتماعات الشراكة مع المريض.

| □ لا أوافق بشدّة |  | □ لا أوافق |  | □ حياديّ |  | □ أوافق |  | □ أوافق بشدّة |
| --- | --- | --- | --- | --- | --- | --- | --- | --- |

1. تم تقديم الدعم لتسهيل مشاركتي في إجتماعات لجنة الشراكة مع المريض. (توجيه, نقل...)

| □ لا أوافق بشدّة |  | □ لا أوافق |  | □ لا أعلم |  | □ أوافق |  | □ أوافق بشدّة |
| --- | --- | --- | --- | --- | --- | --- | --- | --- |

1. كان لديّ معلومات كافية تخولني النقاش في المواضيع المحددة خلال الإجتماع.

| □ لا أوافق بشدّة |  | □ لا أوافق |  | □ لا أعلم |  | □ أوافق |  | □ أوافق بشدّة |
| --- | --- | --- | --- | --- | --- | --- | --- | --- |

مشاركة الآراء خلال الإجتماعات

1. لقد كنت قادراً على التعبير عن آرائي براحة وحريّة تامة.

| □ لا أوافق بشدّة |  | □ لا أوافق |  | □ لا أعلم |  | □ أوافق |  | □ أوافق بشدّة |
| --- | --- | --- | --- | --- | --- | --- | --- | --- |

1. أشعر أن آرائي قد تم سماعها من قبل الفريق.

| □ لا أوافق بشدّة |  | □ لا أوافق |  | □ لا أعلم |  | □ أوافق |  | □ أوافق بشدّة |
| --- | --- | --- | --- | --- | --- | --- | --- | --- |

تأثير وإنعكاسات فعاليات الشراكة مع المريض

1. أعتقد أنه قد تم تحقيق أهداف إجتماعات الشراكة مع المريض.

| □ لا أوافق بشدّة |  | □ لا أوافق |  | □ لا أعلم |  | □ أوافق |  | □ أوافق بشدّة |
| --- | --- | --- | --- | --- | --- | --- | --- | --- |

1. عندي ثقة بأن المعطيات التي قدمتها سيتم أخذها بعين الإعتبار من قبل المستشفى.

| □ لا أوافق بشدّة |  | □ لا أوافق |  | □ لا أعلم |  | □ أوافق |  | □ أوافق بشدّة |
| --- | --- | --- | --- | --- | --- | --- | --- | --- |

1. أعتقد أن المعطيات التي قدمتها خلال الإجتماع ستغير من آليات عمل المستشفى.

| □ لا أوافق بشدّة |  | □ لا أوافق |  | □ لا أعلم |  | □ أوافق |  | □ أوافق بشدّة |
| --- | --- | --- | --- | --- | --- | --- | --- | --- |

1. غير الذي ورد أعلاه, برأيك ما هو تأثير لجنة الشراكة مع المريض؟

□ الإحترام والرعاية عبر تطبيق التواصل العلاجي

□ تحسين التجربة الإستشفائيّة ورضى المرضى

□ مشاركة المريض في صنع القرار

□ غير ذلك

الفوائد والتحديات

1. نتيجة مشاركتي في إجنماعات لجنة الشراكة مع المريض أصبح لديّ معرفة بأنظمة الرعاية الصحيّة والمواضيع ذات صلة.

| □ لا أوافق بشدّة |  | □ لا أوافق |  | □ لا أعلم |  | □ أوافق |  | □ أوافق بشدّة |
| --- | --- | --- | --- | --- | --- | --- | --- | --- |

1. بشكلٍ عام لقد كنت راضٍ عن المشاركة بإجتماعات لجنة الشراكة مع المريض.

| □ لا أوافق بشدّة |  | □ لا أوافق |  | □ لا أعلم |  | □ أوافق |  | □ أوافق بشدّة |
| --- | --- | --- | --- | --- | --- | --- | --- | --- |

1. إن المشاركة بإجتماعات لجنة الشراكة مع المريض يعد إستثماراً جيداً لوقتي الخاص.

| □ لا أوافق بشدّة |  | □ لا أوافق |  | □ لا أعلم |  | □ أوافق |  | □ أوافق بشدّة |
| --- | --- | --- | --- | --- | --- | --- | --- | --- |

1. برأيك ما هي نقاط القوة/الفوائد الناتجة عن إجتماعات لجنة الشراكة مع المريض؟

□ خفض الضيق وتوتر لدى المرضى

□ تحسين التجربة الإستشفائيّة ورضى المرضى

□ تعبير المرضى عن آرائهم

□ تحسين سمعة المستشفى وإنتماء المريض

□ غير ذلك

1. برأيك ما الذي يمكن العمل على تحسينه ما يتعلق بإجتماعات لجنة الشراكة مع المريض؟

□ لا يوجد أي نقاط ضعف

□ توفير الوقت المتاح لحضور الإجتماعات

□ إدارة الحوار خلال الإجتماعات

□ إعطاء مواد تثقيفيّة للمرضى المشاركين

□ غير ذلك

شكراً لك على المشاركة.

**إستبيان إلتزام المريض بأساليب الرعاية الذاتيّة والإبلاغ عن المضاعفات**

إن الهدف من جمع المعلومات هو لبناء قاعدة بيانات عن آليات تقديم الرعاية الطبيّة للمريض وخصيصاً المتعلقة بأسس الشراكة مع المريض خلال مرحلة وضع, تنفيذ وتقييم خطة العلاج. الرجاء الإنتباء الى أننا نحترم ونطبق مبادئ السريّة المهنيّة وأن جميع المعلومات سيتم الإطلاع عليها من قبل المعنيين في المستشفى.

1. الإسم:

**التقييم الصحي لمرضى السرطان**

1. هل أنت مدخن؟ □نعم □ كلا
2. هل تتلقى دعم ومساندة إجتماعيّة من العائلة؟ □نعم □ كلا
3. مسافة النقل من السكن الحالي الى مركز العلاج الكيميائي (المستشفى):

□أقل من 30 دقيقة

□30 – 60 دقيقة

□من ساعة الى ثلاث ساعات

□من ثلاث الى ستة ساعات

□أكثر من ستة ساعات

1. هل يوجد مرضى سرطان ضمن التاريخ الصحي للعائلة: □ نعم □ كلا
2. نوع مرض السرطان:

□سرطان في الدم

□سرطان في الرئة

□سرطان الثدي

□سرطان الجهاز الهضمي والأمعاء

□سرطان في الجهاز التناسلي

□غير ذلك:

1. مرحلة تقدم المرض:

□ مبكر I

□ مبكر II

□ متأخر III

□ متأخر IV

1. هل يوجد أمراض أخرى متزامنة مع مرض السرطان؟ □ نعم □ كلا
2. هل تكرر تشخيص المريض بمرض السرطان؟ □ نعم □ كلا

**خطة العلاج لمريض السرطان**

1. نوع العلاج:

| □علاج كيميائي فقط |
| --- |
| □علاج كيميائي وجراحة |
| □علاج شعاعي فقط |
| □علاج كيميائي, شعاعي وجراحة |
| □علاج شعاعي وجراحة |

1. عدد أنواع العلاج الكيميائي: □ نوع واحد من العلاج كيمائي □ أكثر من نوع علاج كيميائي
2. عدد حلقات العلاج الكيميائي التي خضع لها المريض:

□ الدورة الأولى

□ الدورة الثانية

□ الدورة الثالثة

□ أكثر من ثلاث حلقات من العلاج الكيميائي

1. هل يوجد آثار جانبيّة للعلاج؟ □نعم □ كلا
2. عدد الإستشفاء (دخول المستشفى) بسبب حدوث مضاعفات ناتجة عن العلاج الكيميائي:

□ مرة واحدة

□ مرتين

□ ثلاث مرات أو أكثر

□ ولا مرة

1. نوع التغطية الماليّة للعلاج الكيميائي:

□ وزارة الصحة اللبنانيّة

□ جمعيات ومساعدات صحيّة

□ خاص

□ تأمين أو ضمان

**أنظمة الرعاية الصحيّة (مدى تطبيق أسس الشراكة مع المريض خلال مرحلة صنع القرار الطبي)**

الرجاء إختيار مدى توافق رأيك مع الإحتمالات أدناه المتعلقة بمراحل العلاج الخاصة بك. عبر تحديد رقم على مقياس من 5 الى 1, 5 هو أوافق بشدة و 1 هو لا أوافق بشدّة.

1. برأيي تم إستخدام أسلوب التواصل العلاجي.

|  | لا أوافق بشدّة |  | لا أوافق |  | حياديّ |  | أوافق |  | أوافق بشدّة |
| --- | --- | --- | --- | --- | --- | --- | --- | --- | --- |

1. برأيي أنه تم الإستماع الى وجهات نظري خلال مرحلة العلاج.

|  | لا أوافق بشدّة |  | لا أوافق |  | حياديّ |  | أوافق |  | أوافق بشدّة |
| --- | --- | --- | --- | --- | --- | --- | --- | --- | --- |

1. تم أخذ آرائي بجديّة من قبل الطبيب والفريق الصحي.

|  | لا أوافق بشدّة |  | لا أوافق |  | حياديّ |  | أوافق |  | أوافق بشدّة |
| --- | --- | --- | --- | --- | --- | --- | --- | --- | --- |

1. أعتقد أن آرائي سوف تأثر بالإختيار النهائي لأنواع العلاج الخاصة بي.

|  | لا أوافق بشدّة |  | لا أوافق |  | حياديّ |  | أوافق |  | أوافق بشدّة |
| --- | --- | --- | --- | --- | --- | --- | --- | --- | --- |

1. كان لي أو/ولأفراد العائلة الممثلين قانونياً فرص متساوية مع الطبيب والفريق الصحي للمشاركة بصنع القرار الطبي.

|  | لا أوافق بشدّة |  | لا أوافق |  | حياديّ |  | أوافق |  | أوافق بشدّة |
| --- | --- | --- | --- | --- | --- | --- | --- | --- | --- |

1. أفهم دوري خلال مرحلة العلاج بوضوح.

|  | لا أوافق بشدّة |  | لا أوافق |  | حياديّ |  | أوافق |  | أوافق بشدّة |
| --- | --- | --- | --- | --- | --- | --- | --- | --- | --- |

1. تم توفير معلومات ذات محتوى سهل الفهم بالنسبة لي قبل وخلال مرحلة العلاج ليتم مشاركة رأيي بناءً على معرفة.

|  | لا أوافق بشدّة |  | لا أوافق |  | حياديّ |  | أوافق |  | أوافق بشدّة |
| --- | --- | --- | --- | --- | --- | --- | --- | --- | --- |

1. كنت راضٍ عن المعلومات (التثقيف الصحي) التي قدمها لي الطبيب قبل, خلال وبعد مرحلة العلاج وخلال الحوار مع الطبيب.

|  | لا أوافق بشدّة |  | لا أوافق |  | حياديّ |  | أوافق |  | أوافق بشدّة |
| --- | --- | --- | --- | --- | --- | --- | --- | --- | --- |

1. أعرف أهداف العلاج الخاصة بي.

|  | لا أوافق بشدّة |  | لا أوافق |  | حياديّ |  | أوافق |  | أوافق بشدّة |
| --- | --- | --- | --- | --- | --- | --- | --- | --- | --- |

1. أقوم بإتباع الإرشادات والنصائح الطبيّة المقدمة من الفريق الصحي.

|  | لا أوافق بشدّة |  | لا أوافق |  | حياديّ |  | أوافق |  | أوافق بشدّة |
| --- | --- | --- | --- | --- | --- | --- | --- | --- | --- |

1. أنصح الآخرين بإتباع إرشادات والنصائح الطبيّة المقدمة من الفريق الصحي.

|  | لا أوافق بشدّة |  | لا أوافق |  | حياديّ |  | أوافق |  | أوافق بشدّة |
| --- | --- | --- | --- | --- | --- | --- | --- | --- | --- |

1. برأيك من يجب أن يأخذ القرار المتعلق بالعلاج؟

|  | المريض فقط أو العائلة فقط حين لا يستطيع المريض أخذ القرار |  | قرار مشترك بين المريض والطبيب |  | الطبيب فقط |
| --- | --- | --- | --- | --- | --- |

**أساليب الرعاية الذاتيّة**

1. ما هو معدل قيامك بالمراقبة الذاتيّة للعوارض, التغيرات الجسديّة, تأثير العلاج والمضاعفات التي قد تحدث؟

|  | أبداً |  | بعض الأحيان |  | غالباً |  | دائماً |
| --- | --- | --- | --- | --- | --- | --- | --- |

1. ما هو معدل تغيير نظامك الغذائي؟

|  | أبداً |  | بعض الأحيان |  | غالباً |  | دائماً |
| --- | --- | --- | --- | --- | --- | --- | --- |

1. ما هو معدل تغيير نمط البرنامج الرياضي الخاص بك؟ (مثال ممارسة الرياضة أكثر, التوازن ما بين أوقات الراحة وممارسة النشاط الحركي)

|  | أبداً |  | بعض الأحيان |  | غالباً |  | دائماً |
| --- | --- | --- | --- | --- | --- | --- | --- |

1. ما هو معدل طلب تحصيل دعم من أفراد العائلة أو الأصدقاء؟

|  | أبداً |  | بعض الأحيان |  | غالباً |  | دائماً |
| --- | --- | --- | --- | --- | --- | --- | --- |

1. ما هو معدل طلب تحصيل دعم من الفريق الصحي؟

|  | أبداً |  | بعض الأحيان |  | غالباً |  | دائماً |
| --- | --- | --- | --- | --- | --- | --- | --- |

1. ما هو معدل طلب تحصيل دعم من مرضى سرطان آخرين؟ (مثال حلقة دعم لمرضى السرطان حضوري أو Online)

|  | أبداً |  | بعض الأحيان |  | غالباً |  | دائماً |
| --- | --- | --- | --- | --- | --- | --- | --- |

1. ما هو معدل تقديمك, أو التحضير أو الإنخراط بأنشطة الدعم الإجتماعي لأفراد العائلة والأصدقاء؟

|  | أبداً |  | بعض الأحيان |  | غالباً |  | دائماً |
| --- | --- | --- | --- | --- | --- | --- | --- |

1. ما هو معدل حصر تواصلك الإجتماعي ببعض الأشخاص؟ (مثال الإنعزال الإجتماع, التاصل الإختياري)

|  | أبداً |  | بعض الأحيان |  | غالباً |  | دائماً |
| --- | --- | --- | --- | --- | --- | --- | --- |

1. ما هو معدل محاولتك لتحصيل معلومات عن المرض و/أو العلاج؟

|  | أبداً |  | بعض الأحيان |  | غالباً |  | دائماً |
| --- | --- | --- | --- | --- | --- | --- | --- |

1. ما هو معدل محاولتك لتحصيل معلومات عن الإهتمام بالذات؟

|  | أبداً |  | بعض الأحيان |  | غالباً |  | دائماً |
| --- | --- | --- | --- | --- | --- | --- | --- |

1. ما عو معدل تجاهلك وعدم إهتمامك بالمعلومات الطبيّة؟

|  | أبداً |  | بعض الأحيان |  | غالباً |  | دائماً |
| --- | --- | --- | --- | --- | --- | --- | --- |
